# Beta-lactam, aminoglycoside, and quinolone resistance in *Escherichia coli* strains isolated from shrimps and mussels in the Marmara Sea

**DOI:** 10.17221/105/2022-VETMED

**Published:** 2023-05-29

**Authors:** Baran Celik, Bahar Ergul, Ayse Ilgin Kekec, Baris Hala, Begum Maslak, Belgi Diren Sigirci, Beren Basaran Kahraman, Arzu Funda Bagcigil, Kemal Metiner, Seyyal Ak

**Affiliations:** ^1^Department of Microbiology, Faculty of Veterinary Medicine, Istanbul University – Cerrahpasa, Buyukcekmece/Istanbul, Turkiye; ^2^Ambarli Veterinary Border Control Point Directorate, Republic of Turkiye Ministry of Agriculture and Forestry, Istanbul, Turkiye; ^3^Institute of Graduate Studies, Istanbul University – Cerrahpasa, Avcilar/Istanbul, Turkiye

**Keywords:** aquatic animals, *E. coli*, ESBL, multiple drug resistance

## Abstract

The purpose of the study was to examine the prevalence of *Escherichia coli* in shrimps and mussels, and to determine the distribution of β-lactam, aminoglycoside, quinolone, and multi-drug resistance phenotypically and genotypically in *E. coli* isolates obtained from mussels and shrimps in Istanbul. Faecal samples were collected from mussels (*n* = 96) and shrimps (*n* = 96) from the Marmara Sea coastline and fish markets in Istanbul. For the detection of antibiotic susceptibilities, seven antibiotic groups were used. β-lactamase, aminoglycoside, and quinolone genes were also determined. A total of 34 (17.7%, 15 shrimps, and 19 mussels) *E. coli* were isolated, and 17 (50%) were found to be resistant to one or more antimicrobials. The highest resistance was seen against aminoglycosides with 11 isolates (32.35%), followed by quinolones with 10 isolates (29.41%) and extended-spectrum β-lactamase (ESBL) with 4 isolates (11.76%). Multi-drug resistance was detected in 5 isolates (14.7%) from 3 shrimp and 2 mussel samples. The prevalence of ESBL genes was demonstrated at 3.84% in mussels and shrimp samples. There were no AmpC and carbapenemase-producing genes. These samples harbored *bla*_CTX-M-1_ (*n* = 3) and *bla*_TEM_ (*n* = 4). Ten isolates were resistant to aminoglycosides genotypically. Resistance genes detected were *strB* in 2 isolates, *aadA* in 5, *strB* and *aadA* together in 3, ANT('')-Ia, *aphA1* and *aphA2* simultaneously in 3, *aphA1* in 1, *aac(3)-IIa* in 1 isolate. *aac(6')-Ib-cr* gene was detected in only one of 10 phenotypically resistant isolates to quinolones.

Increasing rates of land-based anthropogenic pollution in marine ecosystems have become an important factor in promoting the emergence of multidrug-resistant (MDR) bacteria in aquatic animals (Kummerer 2009). The rapid spread of *Enterobacteriaceae*, which produce broad-spectrum β-lactamases [such as extended-spectrum β-lactamases (ESBLs), AmpC β-lactamases and metallo-β-lactamases (MBLs)], in coastal marine ecosystems is worrying because enterobacterial species (mostly *E. coli*) are commonly found in the intestinal microbiota of fish (Zhao et al. 2018). Beta-lactam, aminoglycoside, and quinolone are important antibiotic groups used in human and veterinary medicine. Prophylactic or therapeutic applications of antibiotics in aquaculture or pollution can create selective pressure on the natural bacterial population and increase the ability to produce antibiotic-resistant bacteria or resistance genes in the aquatic environment. These bacteria or resistance genes pass from aquatic to terrestrial habitats, and their negative effects on human and animal health are well known. Bacterial resistance to antibiotics is currently recognized as one of the most critical threats to animal health and affects the treatment of infections worldwide (WHO 2018). The study aimed to isolate *E. coli* from shrimps and mussels and to detect the prevalence of ESBL, AmpC beta-lactamase, and carbapenem, aminoglycoside, and quinolone resistance phenotypically and genotypically.

## MATERIAL AND METHODS

### Samples

In the study, 96 shrimp and 96 mussel samples were examined between January and September 2017. The samples were collected from the Marmara Sea coastline (21 mussels) and fish markets (75 mussels and 96 shrimps) in different parts of Istanbul. Each of the shrimp and mussel samples was placed in sterile bags separately. While taking mussel samples, care was taken to ensure that their shells were closed. Using dry ice, the samples were brought to the laboratory immediately.

### Microbiological analysis of isolates

The shrimp samples were dissected through the dorsal part with a sterile scalpel, and the gastrointestinal tract was exposed. The mussel samples were opened from the shell through a sterile scalpel in a frozen state, and a piece of the gastrointestinal tract was removed by a loop. All the faeces from gastrointestinal tracts were planted onto 15 ml of tryptic soy broth (TSB) (HiMedia LQ508) for enrichment and incubated at 37 °C for 24 hours (Overdevest et al. 2011). After the incubation, 10 μl samples from each TSB were planted onto MacConkey agar (HiMedia MH081) with and without cefotaxime (1 mg/l; HiMedia CAS:64485-93-4). They were incubated at 37 °C for 24 hours. Then isolated colonies were examined and confirmed by using standard microbiological methods: Gram staining, oxidase test, IMVIC, and carbohydrate fermentation tests (Quinn et al. 1994).

### Antimicrobial susceptibility testing

The antibiotic susceptibilities tests were performed by the disc diffusion method according to EUCAST and CLSI guidelines (CLSI 2018; EUCAST 2018). For aminoglycoside susceptibilities, amikacin, gentamicin, streptomycin, and kanamycin discs, for quinolone susceptibilities, ciprofloxacin, nalidixic acid, ofloxacin, levofloxacin, and norfloxacin discs were used. For the detection of ESBL, AmpC, and carbapenemase resistance, commercial screening and confirmation kits (Rosco Diagnostica, 98008 and 98015, and 98014) were used. Fresh and pure cultures of suspect isolates were prepared, a 0.5 McFarland density bacterial suspension was prepared from the samples, and 0.1 ml of this suspension was spread on MHA. Cefotaxime (CTX30), Cefotaxime + Clavulanate (CTX+C), Ceftazidime (CAZ30), Ceftazidime + Clavulanate (CAZ+C), Cefepime (FEP30), Cefepime + Clavulanate (FEP+C) antibiotic discs included in the kit were placed on MHA agar. It was incubated at 37 °C for 24 hours. Growth inhibition zone diameters formed at the end of the incubation period were measured and compared with the values given in the kit. The growth inhibition zone of the disks of the cephalosporin group and the zone diameters of the cephalosporin + clavulanate combination disks were compared. The isolate was considered ESBL positive if the cephalosporin combination zone was ≥ 5 mm from the single cephalosporin zone.

Also, for multi-drug resistance, the susceptibilities to tetracycline, chloramphenicol, ampicillin-sulbactam, amoxicillin-clavulanic acid, aztreonam, temocillin and trimethoprim-sulfamethoxazole were used.

### Characterisation of β-lactamase, aminoglycoside, and quinolone resistance genes

As previously described, the presence of β-lac-tamase, quinolone, and aminoglycoside genes was investigated by PCR. The genes used in PCR are given in Table 1. PCR amplifications were done in a Thermal cycler (Axygen Maxygen; Corning Inc., Tewksbury, MA, USA) and visualized in UV Transilluminator (Infinity; Vilber Laurmat, Marne-la-Vallée Cedex, France). The genes and amplification conditions used in PCR are given in Table 1.

**Table 1:** Beta-lactamase, quinolone, and aminoglycoside genes and amplification conditions

Genes	Target groups	Amplification conditions		References
		cycles	number of cycles	
CTX-M multiplex PCR	group 1	94 °C – 5 min	30 cycles	Woodford et al. (2006)
		94 °C – 25 sec		
	group 2	52 °C – 40 sec		
		72 °C – 50 sec		
	group 9	72 °C – 6 min		
				
CTX-M PCR	group 8	94 °C – 5 min	30 cycles	Woodford et al. (2006)
		94 °C – 25 sec		
		52 °C – 40 sec		
	group 25	72 °C – 50 sec		
		72 °C – 6 min		
				
TEM	*bla*_TEM_	94 °C – 3 min	35 cycles	Arlet et al. (1995)
		95 °C – 30 sec		
		55 °C – 30 sec		
		72 °C – 45 sec		
		72 °C – 5 sec		
				
SHV	*bla*_SHV_	95 °C – 5 min	30 cycles	Chanawong et al. (2000)
		95 °C – 15 sec		
		55 °C – 30 sec		
		72 °C – 90 sec		
		72 °C – 5 min		
				
OXA 10	*bla*_OXA-10_	94 °C – 10 min	35 cycles	Vahaboglu et al. (1998)
		94 °C – 60 sec		
		55 °C – 60 sec		
		72 °C – 60 sec		
		72 °C – 10 min		
				
PER	*bla*_PER-2_	95 °C – 3 min	25 cycles	Bauernfeind et al. (1996)
		94 °C – 30 sec		
		60 °C – 30 sec		
		72 °C – 30 sec		
		72 °C – 3 min		
				
AmpC	MOX(1,2), CMY (1-11), *lat* (1-4), *bil*-1, *dha* (1,2), *acc*, *mir*-1 t, *act*-1, FOX(1-5b)	94 °C – 3 min	25 cycles	Perez-Perez and Hanson (2002)
		94 °C – 30 sec		
		64 °C – 30 sec		
		72 °C – 60 sec		
		72 °C – 7 min		
				
OXA 48	*bla*_OXA-48_	94 °C – 10 min	30 cycles	Voets et al. (2011)
		94 °C – 40 sec		
		57 °C – 40 sec		
		72 °C – 60 sec		
		72 °C – 7 min		
				
Quinolone	*qnrA*, *qnrB*, *qnrS*, *qnrC*, *qnrD*, *qepA*, *aac(6')-Ib-cr*	95 °C – 15 min	30 cycles	Ciesielczuk et al. (2013)
		94 °C – 30 sec		
		63 °C – 40 sec		
		72 °C – 90 sec		
		72 °C – 10 min		
				
Aminoglycosides	*strA*, *strB*, *aph(3)-Ia*, *aac(3)-IIa*, *aac(3)-III*, *aac(3)-IV*, *aac(6')-Ib*, *ant(3')-Ia*, *aadA*, *aphA1*, *aphA2*, *ANT(2'')-Ia*, *armA*, *armB*	94 °C – 3 min	35 cycles	Vakulenko et al. (2003)
		94 °C – 40 sec		
		55 °C – 40 sec		
		72 °C – 40 sec		
		72 °C – 2 min		

## RESULTS

### Bacterial isolates and antimicrobial susceptibility testing

A total of 34 (17.7%, 15 shrimps, and 19 mussels) *E.* *coli* were isolated from MacConkey agar, and only two (1.04%) strains were grown on MacConkey agar-containing cefotaxime. All isolates were tested for beta-lactams, AmpC, and carbapenem and their antibiotic susceptibilities to the above-mentioned antibiotics were determined. Those two isolates isolated from MacConkey with cefotaxime, isolated from shrimps (2/15, 13.3%) and were confirmed as ESBL producers. There was no AmpC beta-lactamase, and carbapenem resistance was detected. However, one (2.94%) of the isolates obtained from shrimp was seen as temocillin resistant.

**Figure 1:**
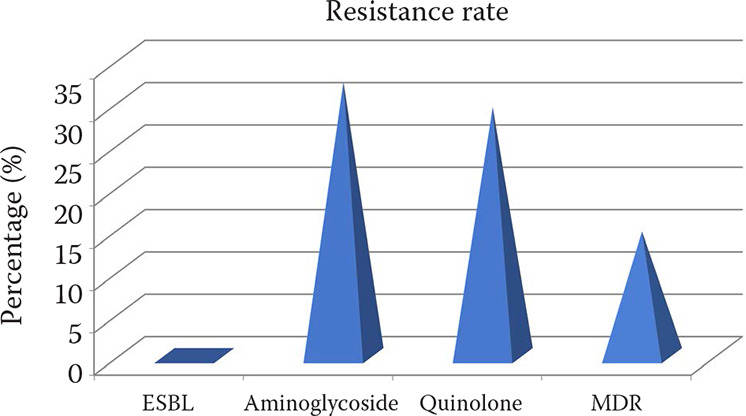
The results of the resistance rate of the *E. coli* isolates ESBL = extended-spectrum β-lactamase; MDR = multidrug resistance

Half of the isolates (17/34) were resistant to one or more antimicrobials. Phenotypic resistance was detected as follows. Six (17.6%) isolates were resistant to penicillin, 6 (17.6%) isolates were resistant to sulphonamide, 5 (14.7%) isolates were resistant to phenicol, 6 (17.6%) isolates were resistant to tetracycline, 11 (32.35%) isolates were resistant to aminoglycosides, and 10 (29.41%) isolates were resistant to quinolones. MDR was detected in five isolates (14.7%) from three shrimp and two mussel samples. One (2.94%) of the isolates was resistant to 3, two (5.88%) to 5, and two (5.88%) to 6 antibiotic classes. The results of the resistance rate of the *E.* *coli* isolates are shown in Figure 1 and the phenotypic and genotypic resistance rates and patterns are summarised in Table 2.

**Table 2:** Phenotypic and genotypic resistance patterns

Source	Phenotype pattern	Genotype pattern
Mussel imported	SAM, AMC, AK	–
Shrimp imported	NOR	–
Mussel imported	S, K, CIP, AN, OFX, NOR	*strB*, *aphA1*
Mussel imported	S, CIP, NA	*strB*, CTX-M-1 group, *bla*_TEM_
Mussel domestic	SAM, SXT	*bla*_TEM_
Mussel domestic	S	*aadA*
Shrimp domestic	NOR	–
Mussel domestic	S, CIP, NA, OFX, LEV, NOR	*aadA*
Shrimp domestic	TE, S	*aadA*
Shrimp domestic	TE, S	*aadA*
Mussel domestic*	SXT, C, TE, S, K, NA	*aadA*
Mussel domestic	OFX, LEV	–
Shrimp domestic	TEMOC	–
Mussel domestic*	SXT, C, TE	–
Shrimp domestic*	SAM, SXT, C, S, K, NA	*strB*, *aad*A, ANT(2'')Ia, *aphA1*, *aphA2*
Shrimp domestic*	CTX, CAZ, FEP, SAM, ATM, SXT, C, TE, N, S, K, CIP, NA, OFX, LEV, NOR	*strB*, *aadA*, ANT(2'')-Ia, *aphA1, aphA2*, CTX-M-1 group, *bla*_TEM_
Mussel domestic*	CTX, CAZ, FEP, SAM, ATM, SXT, C, TE, CN, S, K, CIP, NA, OFXLEV, NOR	CTX-M-1 group*, bla*_TEM_, *aac(3)-IIa*, *strB*, *aadA*, ANT(2'')-Ia, *aphA1*, *aphA2*, *aac(6')-Ib-cr*

*Multidrug-resistant strains: Phenotypical ESBL

AK = amikacin (30 μg); AMC = amoxicillin-clavulanic acid (30 μg); ATM = aztreonam (30 μg); C = chloramphenicol (30 μg); CAZ = ceftazidime; CIP = ciprofloxacin (5 μg); CN = gentamicin (10 μg); CTX = cefotaxime; FEP = cefepime; K = kanamycin (30 μg); LEV = levofloxacin (5 μg); MRP = meropenem; NA = nalidixic acid (30 μg); NOR = norfloxacin (10 μg); OFX = ofloxacin (5 μg); S = streptomycin (10 μg); SAM = ampicillin-sulbactam (20 μg); SXT = trimethoprim-sulfamethoxazole (25 μg); TE = tetracycline (10 μg); TEMOC = temocillin

### Characterisation of resistance genes by PCR

ESBL resistance gene was genotypically detected in four isolates, whereas only two were phenotypically confirmed as ESBL. The distribution of the ESBL and beta-lactamase genes was *bla*_TEM_ and *bla*_CTX-M-1_. The two genes (*bla*_CTX-M_ + *bla*_TEM_) were found to co-occur in three isolates; the *bla*_TEM_ gene was harboured from one isolate. As mentioned in the carbapenemase screening test, the isolate, which was thought to have the OXA-48 gene because it was resistant to temocillin, could not be confirmed by PCR analysis.

Ten (90.9%) of the 11 phenotypically resistant isolates to aminoglycosides were also resistant genotypically. Resistance genes were detected in all ten isolates, which were resistant to streptomycin [*strB* in two (18%) isolates, *aadA* in five (45.45%), *strB* and *aadA* together in three (27.27%)]. Of the five phenotypically kanamycin-resistant isolates, four (80%) had resistance genes [ANT(2'')-Ia, *aphA1,* and *aphA2* simultaneously in 3 (60%) isolates and *aphA1* in one (20%)]. Also, *aac(3)-IIa* was detected in only one (50%) of the two isolates phenotypically resistant to gentamicin. One isolate that was phenotypically resistant to amikacin was not found resistant genotypically. Quinolone resistance gene, *aac(6')-Ib-crm*, was detected in only one (10%) of 10 phenotypically resistant isolates. Genotypic resistance patterns are summarized in Table 2.

## DISCUSSION

The World Health Organization (WHO) defines antibiotic resistance as one of the greatest threats to global health, food security, and development (WHO 2021). Since 2000, the problem of antibiotic resistance, especially ESBL and/or AmpC-producing *E. coli*, has been spreading rapidly worldwide and is becoming an important problem with its increasing medical importance (Obeid et al. 2018). In many studies, ESBL, aminoglycoside, quinolone, and multi-drug resistance have been reported in *E. coli* strains isolated from various animals (Rashid et al. 2015; Kahraman et al. 2016; Gumus et al. 2017; Pehlivanoglu 2019; Palma et al. 2020). However, there are limited studies on the bivalve, gastropods, molluscs, and crustacean shellfish (Reverter et al. 2020; Sharma et al. 2021).

According to the study conducted by Noor Uddin et al. (2013), *E. coli* does not exist in the gastrointestinal system flora of water products; however, Gerzova et al. (2014) and Brahmi et al. (2018) believe the existence of *E. coli* in fish microbiota. Sullam et al. (2012) and Zhao et al. (2018) revealed that the intestinal microbiota of the fish might vary depending on factors such as nutrition, travel distance, existence in a contaminated area, and immune system. Researchers indicated the presence of *E. coli* in aquatic environments as an indicator of faecal contamination (Devane et al. 2020; Pendergraph et al. 2021).

In Seoul, Korea, 179 (6.7%) *E. coli* isolates were obtained (57 from 834 shellfish, 74 from 971 fish, 41 from 652 molluscs, and 7 from 206 crustaceans) (Ryu et al. 2012). In a study conducted in Vietnam, 20 *E. coli* were isolated from shellfish, and the *bla*_TEM_ gene was detected in 5 isolates (Van et al. 2008). Singh et al. (2020) reported that 79 (52.66%) of 150 *E. coli* isolates obtained from shellfish were ESBL. In a study conducted in Algeria, three (8.8%) of 34 Gram-negative bacterial isolates from seawater were positive for ESBL (Alouache et al. 2012). In another study conducted in Algeria, 21.3% ESBL positivity was found in Gram-negative bacteria isolated from the intestines and gills of 300 fish (Brahmi et al. 2018). It was determined that the ESBL positivity rate was 50% in 8 *E. coli* isolates obtained from water samples in Bangladesh (Rashid et al. 2015). *E. coli* was isolated from 42 fish caught from the wild habitats of the Atlantic coast of Brazil, and it was reported that the CTX-M gene, which causes ESBL positivity, was found in this region for the first time (Sellera et al. 2018). In our country, Kurekci et al. (2017) investigated ESBL-positive *E. coli* in water samples taken from the Asi River and its nearby wastewater treatment plant. They determined 54 ESBL-positive isolates and concluded that surface waters in Turkiye are an aquatic reservoir for transporting ESBL genes. In this study, 34 *E. coli* were isolated from shrimp and mussels, and 5.88% (2/34) were identified as ESBL-producing *E. coli* phenotypically and 11.76% genotypically. The prevalence of ESBL-producing *E. coli* in domestic shrimps was 2.08% (2/96). There is no *E. coli* in the natural microbiota of shrimps, but this study showed they could carry AMR coliform bacteria. For this reason, it is thought that they are considered important for human health, as they spend their lives in coastal areas during the summer months. In addition, the prevalence of ESBL-producing *E. coli* in humans, animals, and the aquatic environment suggested that the presence of resistant bacteria in the microbiota of fishery products is important.

Ng et al. (2018) reported that 81.8% ESBL was detected, including 32 ESBL and 1 KPC *E. coli* isolate. The strains were reported to be resistant to ampicillin (100.0%), cefazolin (100.0%), ceftazidime (97.0%), cefepime (97.0%), and ampicillin/sulbactam. The *sul1, qnrA, bla*_OXA_ 101, *sul2,* and *bla*_CTX-M_ 102 genes were found in the isolates. In the study of Divya et al. (2020), *bla*_TEM_ was detected in 50% of shellfish isolates. Dib et al. (2018) reported the prevalence of *E.* *coli* in shrimps as 66% in northeastern Algeria and stated that none of the *E. coli* isolates had *bla*_OXA_ or *bla*_SHV_ genes. However, phenotypically, all five strains of ESBL were found to harbor the *bla*_CTX-M-15_ gene. In another study on oysters in Amazonia, the prevalence of *E. coli* with a single ESBL phenotype was reported as 17.31%, and the *bla*_TEM_ gene was revealed (Oliveira et al. 2020). In the current study, the genotypically ESBL resistance gene was found in four isolates. Two of the isolates were found to be ESB-positive phenotypically. The distribution of ESBL genes was determined as *bla*_TEM_ and *bla*_CTX-M-1_. The ESBL gene *bla*_CTX-M_ and *bla*_TEM_ were detected together in two of the three isolates; the *bla*_TEM_ gene was also found in one isolate. The isolate, which was thought to have the OXA-48 gene because it was resistant to temocillin in the carbapenemase screening test, could not be confirmed by PCR analysis. Although contamination levels are low, the presence of ESBL-producing and MDR-positive *E. coli* in seafood is assumed to be important to public health due to the potential for human transmission from seafood consumption. To the best of our knowledge, this study demonstrated for the first time that multiple ESBL genes are harbored in *E. coli* isolated from shellfish. This result showed that the *bla*_CTX-M_ gene of the plasmid could be transferred to the aquatic environment. All this highlights the potential hazard associated with seafood from the Sea of Marmara in Istanbul that contains MDR-positive *E. coli* and produces ESBL.

Most antibiotics are released into the environment in various ways due to poor absorption and decomposition by humans and animals (Kummerer 2009), which has made antibiotic pollution a worrying problem. Because aquatic animals are vulnerable to external influences due to their unique habitats, the guts of marine animals have often been recognised as an important niche for transferring antibiotic resistance genes (Zhao et al. 2018).

As a result, the unnecessary use of antibiotics in aquaculture has become a growing problem for human and animal health and the environment. Kumar et al. (2005) obtained 73 *E. coli* isolates (38.8%) from 188 fish, water, and ice samples in India. They treated these isolates with 14 different antibiotics and detected multiple antibiotic resistance by 34.2% (*n* = 25). In Italy, Foti et al. (2009) obtained 57 Gram-negative bacteria isolates from oral and cloacal swaps of 19 sea turtles (*Caretta caretta*). They evaluated multiple antibiotic resistance profiles by treating the isolates with 31 different antimicrobic agents. They reported that at least all the isolates were resistant to one antibiotic. In contrast, more than half of the isolates were resistant to 9 antibiotics. Multiple drug resistance was detected in several *E. coli* isolates at a significant level along with other bacteria species. Dib et al. (2018) showed that *E. coli* isolated from shrimps were completely (100%) resistant to ampicillin, amoxicillin, cephalothin, amikacin, kanamycin, gentamicin, neomycin, and tobramycin.

Akkan et al. (2015) obtained 78 *E. coli* isolates from water samples in fish farms on Iskenderun Gulf and addressed higher multiple resistance profiles. The multi-antibiotic resistance of the isolates in this study was found to be 2.6% (5/192). Five of 34 (14.7%) isolates were resistant to three or more antibiotic groups; antibiotic resistance rates were 32.35% to aminoglycosides, 29.41% to quinolones, 17.6% to sulfonamides, 17.6% to tetracyclines and 14.7% to phenicol. It is essential that the shrimp and mussels used in the research are from their natural (wild) habitats, have not directly encountered any therapeutic agents, and carry a multi-resistant bacterium that is not in the normal microbiota of the creature. Detection of multiple antibiotic resistance in these organisms consumed as food poses a risk to human health.

Reports on aminoglycoside resistance in *E. coli* have been made from many countries such as France, Japan, Taiwan, Spain, South Korea, Belgium, and China. In the study conducted by Tadesse et al. (2012) on 1 729 *E. coli* isolates in the USA between 1950 and 2002, aminoglycoside resistance was investigated phenotypically, and the resistance rates were 0.1% in humans (*n* = 983) and 16.1% in cattle (*n* = 323), 16.7% in chicken (*n* = 138), 14% (*n* = 285) in the pig. In Seoul, Korea, 179 (6.7%) *E. coli* isolates were obtained from 834 shellfish, and it was reported that 5.6% were resistant to nalidixic acid and 5% to kanamycin. The aminoglycoside resistance gene *aadA*, detected in 18 resistant isolates, was found in 16 of 23 streptomycin-resistant isolates, while the presence of the gene was detected in 2 streptomycin-susceptible isolates (Ryu et al. 2012). Lim et al. (2018) found the prevalence of resistance in the *strA-strB* gene of 40 isolates resistant to streptomycin to be 77.5% in their study from 269 *E. coli* isolates obtained from shellfish samples. The prevalence of *the aadA* gene, the most common determinant of streptomycin resistance, was 30%. While *aadA* and *strA-strB* determinants were found simultaneously in six isolates (15%), it was stated that three of the resistant isolates (7.5%) did not have both resistance determinants examined in the study. In Turkiye, aminoglycoside resistance was determined as 4.46% in the gills and 5.53% in the intestines of fish (Ozcan and Balik 2008). In another study, it was determined as 1.1% in the gills and 4% in the intestines of fish (Matyar et al. 2009). Of 34 *E. coli* isolates obtained in this study, 29.4% were resistant to streptomycin, 14.7% to kanamycin, 5.8% to gentamicin, and 2.9% to amikacin. Streptomycin resistance genes were detected in all ten isolates resistant to streptomycin (two isolates with *strB*, five isolates with *aadA,* and three isolates with *strB* and *aadA* together). Resistance genes were found in four of the five isolates that were phenotypically resistant to kanamycin [ANT(2'')-*Ia* and *aphA2* simultaneously in 3 isolates and *aphA1* in one isolate]. The *aac(3)-IIa* gene was detected in only one of the two phenotypically resistant to gentamicin isolates. An isolate that was phenotypically resistant to amikacin was not found to be genotypically resistant. When aminoglycoside-resistant *E. coli* isolates were compared with other antibiotic groups used in the study, it was noted that the highest resistance developed against aminoglycoside. In a search conducted on Gram-negative bacteria isolated from seawater samples in Algeria, 5.8% were resistant to ciprofloxacin and 85.2% to nalidixic acid (Alouache et al. 2012). Jiang et al. (2012) examined 300 farmed fish samples in China and found resistance to ciprofloxacin in 73.8% of the 80 *E. coli* isolates. In a study conducted in the same country by Zhao and Dang (2012) on surface seawater samples, nalidixic acid resistance was found in 82 Gram-negative strains at a rate of 66%. In the study conducted by Boss et al. (2016) on salmon, panga fish, shrimp, and oysters in Switzerland, 22% of 60 *E. coli* isolates were found resistant to ciprofloxacin and 12% to nalidixic acid. In the first study investigating plasmid-mediated quinolone resistance in an environmental bacterium in Turkiye, Ozgumus et al. (2009) detected a quinolone resistance gene in one (1.1%) of 88 *E. coli* isolates obtained from different river waters on the North East coastline. In this study, ten quinolone-resistant strains were found, and the resistance rate was determined as 29.4%. The *aac(6')Ib-cr* gene was detected in only one of 10 phenotypically resistant to quinolones isolates. The previously reported prevalence of antibiotic-resistant bacteria and resistant *E. coli* genes in shellfish is considered highly variable as it is influenced by determinants such as anthropogenic contamination (different antibiotic use and wastewater treatment), water hygiene, processing and packaging hygiene, and market hygiene. This ratio was significant compared to studies conducted in our country and other countries.

Isolation of *E. coli,* which is not a normal microbiota agent by 17.7%, ESBL positivity by 1.04%, and multiple drug resistance by 14.7%, are considered an indicator of exposure of the sea ecosystem to resistant bacteria through wastes and faecal contamination. Detection of *E. coli* with ESBL activity in shrimps caught in the Marmara Sea and multi-antibiotic resistant *E. coli* in shrimps and mussels caused a water reservoir to be formed with these creatures.

Consequently, the gradual increase in antibiotic resistance has become a threatening factor for human and animal health as well as for the ecosystem. The *E. coli* with animal-originating antibiotic resistance is a donor source for other pathogenic *E. coli* strains. Since *E. coli* with ESBL activity was detected phenotypically in the shrimps caught in the Marmara Sea, the existence of *E. coli* with multiple antibiotic resistance within the shrimps and mussels caused the formation of an aquatic reservoir. Such hazards for human consumption should be discussed since this danger is not only a reality for human consumption, but also caused or will cause the transmission and spread of resistant genes with the participation of fish, birds living in the region, and migratory birds and mammals in the food chain.
